# Identification of a putative α-galactoside β-(1 → 3)-galactosyltransferase involved in the biosynthesis of galactomannan side chain of glucuronoxylomannogalactan in *Cryptococcus neoformans*

**DOI:** 10.3389/fmicb.2024.1390371

**Published:** 2024-05-22

**Authors:** Chihiro Kadooka, Yutaka Tanaka, Daisuke Hira, Takuji Oka

**Affiliations:** ^1^Department of Biotechnology and Life Sciences, Faculty of Biotechnology and Life Sciences, Sojo University, Kumamoto, Japan; ^2^Division of Infection and Host Defense, Tohoku Medical and Pharmaceutical University, Sendai, Japan

**Keywords:** *Cryptococcus neoformans*, galactosyltransferase, glucuronoxylomannogalactan, polysaccharide, virulence factor

## Abstract

The cell surface of *Cryptococcus neoformans* is covered by a thick capsular polysaccharide. The capsule is the most important virulence factor of *C. neoformans*; however, the complete mechanism of its biosynthesis is unknown. The capsule is composed of glucuronoxylomannan (GXM) and glucuronoxylomannogalactan (GXMGal). As GXM is the most abundant component of the capsule, many studies have focused on GXM biosynthesis. However, although GXMGal has an important role in virulence, studies on its biosynthesis are scarce. Herein, we have identified a GT31 family β-(1 → 3)-galactosyltransferase Ggt2, which is involved in the biosynthesis of the galactomannan side chain of GXMGal. Comparative analysis of GXMGal produced by a *ggt2* disruption strain revealed that Ggt2 is a glycosyltransferase that catalyzes the initial reaction in the synthesis of the galactomannan side chain of GXMGal. The *ggt2* disruption strain showed a temperature-sensitive phenotype at 37°C, indicating that the galactomannan side chain of GXMGal is important for high-temperature stress tolerance in *C. neoformans*. Our findings provide insights into complex capsule biosynthesis in *C. neoformans*.

## 1 Introduction

*Cryptococcus neoformans*, a basidiomycete yeast, is the primary pathogen responsible for cryptococcosis, a globally prevalent disease that affects immunocompromised individuals, specifically those infected with HIV (Maziarz and Perfect, 2016; Altamirano et al., 2020). Upon pulmonary infection, *C. neoformans* disseminates to the central nervous system and causes severe meningoencephalitis with a high mortality rate.

Capsular polysaccharides, specifically glucuronoxylomannan (GXM), glucuronoxylomannogalactan (GXMGal), and a small amount of mannoproteins, are the key virulence factors of *C. neoformans* (Bose et al., 2003). Several acapsular mutants of *C. neoformans* have been shown to nearly completely lack pathogenicity in mice models, suggesting that enzymes involved in capsule biosynthesis are targets for developing antifungal drugs against cryptococcosis (O’Meara and Alspaugh, 2012; Casadevall et al., 2019). The detailed structures of GXM and GXMGal have been determined. GXM is a larger polysaccharide (MW: 1,000,000–7,000,000) consisting of α-mannan core chains of α-(1 → 3)-linked mannose (Man) and β-linked glucuronic acid (GlcA) and xylose (Xyl) (Cherniak et al., 1998). GXMGal is a relatively smaller polysaccharide (MW: ~100,000), consisting of α-galactan core chains of α-(1 → 6)-linked galactose (Gal) with galactomannan side chains (Vaishnav et al., 1998). Variable numbers of Xyl and GlcA residues are attached to the galactomannan side chain (Heiss et al., 2009). Galactofuranose (Gal*f*) is also found attached to the α-galactan backbone (Heiss et al., 2013). Man residues in GXM and GXMGal are *O*-acetylated; in GXMGal, 80% of the Man residues in the galactomannan side chain are acetylated, influencing the immunomodulatory and immunogenic properties of this polysaccharide (Previato et al., 2017).

GXM is present in ~90% of the capsules, whereas GXMGal is present in only 7–10% of the capsules (Doering, 2009). Thus, although there is a large difference in the amounts of GXM and GXMGal, mutants lacking either polysaccharide lose virulence (Chang and Kwon-Chung, 1994; Moyrand et al., 2007; Kumar et al., 2011). The role of UDP-Gal is crucial, which is evident as disruptants of the genes *UGE1*, encoding UDP-glucose 4-epimerase, and *UGT1*, encoding a Golgi-localized UDP-Gal transporter, are avirulent, likely due to a deficiency in GXMGal (Moyrand et al., 2008; Li et al., 2017). This indicates that galactosyltransferases involved in Golgi-localized GXMGal biosynthesis are targets for antifungal drug development. However, GXMGal biosynthesis-related glycosyltransferases are limited to only two species, β-(1 → 2)-xyltransferases Cxt1 and Cxt2 (Klutts et al., 2007; Klutts and Doering, 2008; Reilly et al., 2009; Wang et al., 2018). The putative α-Gal β-(1 → 3)-Gal transferase Pvg3 in *Schizosaccharomyces pombe*, which belongs to the GT31 family, highlights the diversity of glycosyltransferases (Andreishcheva et al., 2004; Fukunaga et al., 2023). In *C. neoformans*, the genome encodes three GT31 family glycosyltransferases, one of which, Ggt1, functions as an α-Man β-(1 → 6)-Gal transferase in GIPC biosynthesis (Wohlschlager et al., 2013). However, the functions of other presumptive glycosyltransferases of the GT31 family in the *C. neoformans* genome are unclear.

Herein, we report that a putative glycosyltransferase belonging to the GT31 family in *C. neoformans* is involved in GXMGal biosynthesis. Using the *cap59* disruption strain (a GXM-deficient strain of *C. neoformans*) as the parental strain, we constructed disruption strains of GT31 family glycosyltransferases (*ggt1*, *ggt2*, and *ggt3*). A nuclear magnetic resonance and methylation gas chromatography–mass spectrometry analysis of the structure of GXMGal produced by the *cap59* and *ggt2* double-disruptant strain revealed that the galactomannan side chain was reduced or almost completely lost. The *ggt2* disruption strain exhibited a temperature-sensitive (Ts) phenotype at 37°C. These results indicate that, in *C. neoformans*, the galactomannan side chain of GXMGal has an important role in high-temperature stress tolerance.

## 2 Materials and methods

### 2.1 Strains and medium

The *C. neoformans* strains used in this study are listed in Supplementary Table S1. The *C. neoformans* var. *grubii* H99 strain was obtained from the Fungal Genetics Stock Center (Kansas City, USA).

The strains were cultured on YPD medium (2% w/v glucose, 2% w/v peptone, and 1% w/v yeast extract). To produce GXMGal, the strains were cultured in 10% Sabouraud liquid medium [0.4% w/v glucose, 0.1% w/v peptone, 0.1% w/v tryptone, and 50 mM 3-morpholinopropanesulfonic acid (pH 7.3)].

### 2.2 Construction of the *cap59* disruption strain

*CAP59* (CNAG_00721) was disrupted in *C. neoformans* H99 by inserting *NEO* using the CRISPR/Cas9 system (Huang et al., 2022). A gene replacement cassette encompassing a 50-bp homology arm at the 5′ end and a 50-bp homology arm at the 3′ end of *CAP59* was amplified by recombinant PCR using pNEO_6xHA (So et al., 2017) as a template and the cap59-del-F–cap59-del-R primer pair (Supplementary Table S2). The Cas9 expression cassette was amplified by PCR using pBHM2403 as a template and the M13-F–M13-R primer pair. The sgRNA expression cassette was amplified in two PCR steps. An sgRNA scaffold containing a 20-bp target sequence and U6 promoter and an sgRNA scaffold containing a 20-bp target sequence and U6 terminator was amplified by PCR using pBHM2329 as a template and the M13-F–cap59-gRNA-R1 and cap59-gRNA-F2–M13-R primer pairs. The PCR fragments were combined by fusion PCR using the U6-F–U6-R primer pair. All PCR fragments were introduced into *C. neoformans* by electroporation using Gene Pulser II (Bio-Rad, Hercules, CA), yielding *cap59*Δ strain. Transformants were selected using YPD agar plates supplemented with 200 μg/mL G418. The introduction of *NEO* into each locus was confirmed by PCR using the cap59-comf-F–cap59-comf-R primer pair (Supplementary Figure S1).

### 2.3 Construction of the *ggt1*, *ggt2*, and *ggt3* disruption strains

*GGT1* (CNAG_01385), *GGT2* (CNAG_01050), and *GGT3* (CNAG_06918) were disrupted in *C. neoformans* H99 and *cap59*Δ strains by inserting *HYG*. Gene replacement cassettes encompassing a 50-bp homology arm at the 5′ end and a 50-bp homology arm at the 3′ end of *GGT1*, *GGT2*, and *GGT3* were amplified by recombinant PCR using pHYG_GFP (So et al., 2017) as a template and the xxxx-del-F–xxxx-del-R (where “xxxx” indicates *GGT1*, *GGT2*, or *GGT3*) primer pair (Supplementary Table S2). An sgRNA scaffold containing a 20-bp target sequence and U6 promoter and an sgRNA scaffold containing a 20-bp target sequence and U6 terminator were amplified by PCR using pBHM2329 as a template and the M13-F–xxxx-gRNA-R1 and xxxx-gRNA-F2–M13-R primer pairs. All PCR fragments were introduced into *C. neoformans* H99 and *cap59*Δ by electroporation using Gene Pulser II (Bio-Rad, Hercules, CA), yielding *ggt1*Δ, *ggt2*Δ, *ggt3*Δ, *cap59*Δ*ggt1*Δ, *cap59*Δ*ggt2*Δ, and *cap59*Δ*ggt3*Δ strains. Moreover, transformants were selected on YPD agar plates supplemented with 200 μg/mL hygromycin B. The introduction of *HYG* into each locus was confirmed by PCR using the xxxx-comf-F–xxxx-comf-R primer pair (Supplementary Figure S1).

### 2.4 Construction of the *cap59*, *ggt2*, and *ggt3* triple disruption strain

*GGT3* was disrupted in *C. neoformans cap59*Δ*ggt2*Δ strain by inserting *NAT*. A gene replacement cassette encompassing a 50-bp homology arm at the 5′ end and a 50-bp homology arm at the 3′ end of *GGT3* was amplified by recombinant PCR using pNAT_mCherry (So et al., 2017) as a template and the ggt3-del-F–ggt3-del-R primer pair (Supplementary Table S2). Transformants were selected on YPD agar plates supplemented with 100 μg/mL nourseothricin sulfate. The introduction of *NAT* into each locus was confirmed by PCR using the ggt3-comf-F–ggt3-comf-R primer pair (Supplementary Figure S2).

### 2.5 Complementation of the *ggt2* disruption strain with wild-type *GGT2*

For complementation, analysis of *GGT2* using a gene replacement cassette, encompassing a homology arm at the 5′ end of *GGT2*, wild-type *GGT2* containing 3′-UTR, hygromycin B resistance gene (*hph*), and a homology arm at the 3′ end of *GGT2*, was constructed by recombinant PCR using H99 genomic DNA; pNAT_mCherry as a template; and the ggt2-comp-1–ggt2-comp-2, ggt2-comp-3–ggt2-comp-4, and ggt2-comp-5–ggt2-comp-6 primer pairs. The resultant DNA fragment was amplified with the ggt2-comp-1–ggt2-comp-6 primer pair. An sgRNA scaffold containing a 20-bp target sequence and U6 promoter and an sgRNA scaffold containing a 20-bp target sequence and U6 terminator were amplified by PCR using pBHM2329 as a template and the M13-F–HYG-gRNA-R1 and HYG-gRNA-F2–M13-R primer pairs. All PCR fragments were introduced into *C. neoformans ggt2*Δ and *cap59*Δ*ggt2*Δ by electroporation. Transformants were selected on YPD agar plates supplemented with 100 μg/mL nourseothricin sulfate. The introduction of *NAT* into each locus was confirmed by PCR using the ggt3-comp-comf-F–ggt3-comp-comf-R primer pair (Supplementary Figure S1).

### 2.6 Measurement of capsule size

*C. neoformans* strains were cultured in 3 mL of YPD liquid medium at 30°C for 24 h. The cells were then collected by centrifugation and washed three times with sterile phosphate-buffered saline (PBS), suspended in 2 mL of 10% Sabouraud liquid medium, and incubated at 30°C for 24 h to induce capsule production. The culture medium was diluted with PBS mixed at a ratio of 1:1 with India ink (Syogeikuretake Shikon BB1-18; Kuretake Co., Ltd. Nara, Japan) and incubated for 15 min. Images of stained cells were acquired using a microscope equipped with a digital camera. The diameters of the cells and capsules were measured immediately (50 cells), and the average diameter was calculated.

### 2.7 Preparation of the GXMGal fraction

Purification of GXMGal was performed as described (Rocha et al., 2015). Briefly, *cap59*Δ strains were cultivated in 1 L of 10% Sabouraud medium at 30°C with shaking (160 rpm) for 5 days. The culture supernatant was collected by centrifugation, mixed with an equal volume of phenol:chloroform, and centrifuged. The collected supernatant was dialyzed overnight at 4°C using a Visking Tube (Nihon Medical Science, Inc. Japan). The polysaccharides were powdered by lyophilization. GXMGal powder was dissolved in 3% cetyltrimethylammonium bromide solution in 1% borate at pH 9.5. The GM fraction was collected as precipitate, washed with 75% ethanol, dialyzed with water, and lyophilized.

### 2.8 Methylation GC–MS and nuclear magnetic resonance spectroscopy

Glycosidic linkages were analyzed as previously described (Klutts and Doering, 2008; Katafuchi et al., 2017). Briefly, GXMGal was separately dissolved in dimethyl sulfoxide, followed by NaOH addition. After stirring for 3 h, methyl iodide was added, and the suspension was stirred for 24 h. The methylated products were extracted in chloroform and washed using dH_2_O. Then, the methylated samples were hydrolyzed using 2 M trifluoroacetic acid, reduced, and acetylated. The partially methylated alditol acetates were analyzed by GC–MS using a capillary column (30 m × 0.25 mm; DB-5, Agilent, CA) with helium as the carrier gas at a gradient temperature program of 210°C–260°C at 5°C/min. The GC–MS analyses were performed using a JMS-K9 mass spectrometer (JEOL, Tokyo, Japan). NMR experiments were performed as previously described (Klutts and Doering, 2008; Katafuchi et al., 2017). The NMR spectra were recorded using a JNM-LA600 spectrometer (JEOL) at 45°C. Proton and carbon chemical shifts were referenced relative to internal acetone at δ 2.225 and 31.07 ppm, respectively.

## 3 Results

### 3.1 Identification of candidate galactosyltransferases involved in GXMGal biosynthesis in *C. neoformans*

**Figure 1:**
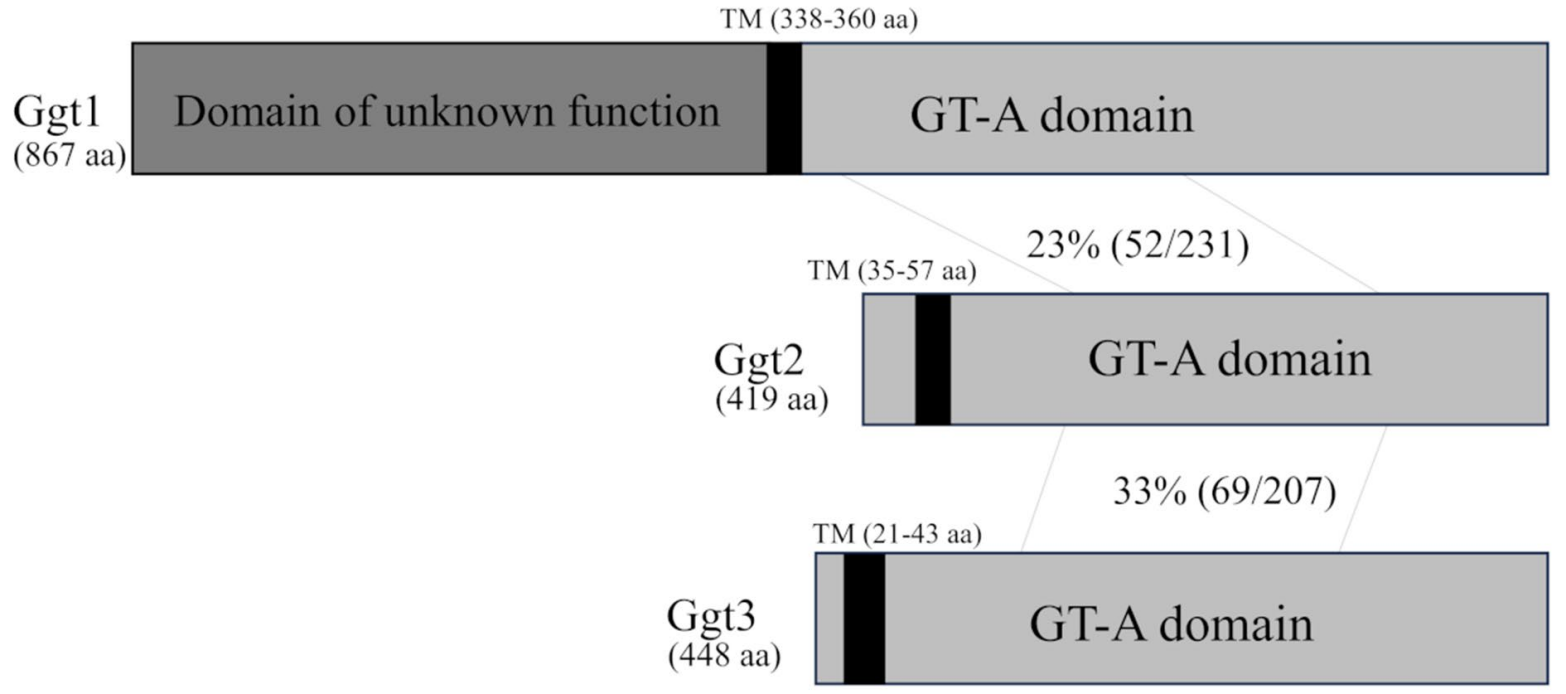
Schematic of the Ggt1, Ggt2, and Ggt3 proteins. The vertical black bars indicate transmembrane (TM) domains of Ggt1 (338–360 aa), Ggt2 (35–57 aa), and Ggt3 (21–43 aa). The gray bars indicate GT-A fold domains of Ggt1, Ggt2, and Ggt3. The dark gray bar indicates an unknown domain of Ggt1. The identity of the amino acid sequences of Ggt1 and Ggt2 and Ggt2 and Ggt3 in the GT-A fold domain are indicated.

First, we searched the *C. neoformans* H99 genome for candidate genes encoding enzymes that transfer the β-galactosyl residue to the hydroxyl group at position 3 of the α-galactosyl residue in GXMGal. *S. pombe* Pvg3 is a glycosyltransferase that exhibits similar enzymatic activity. Therefore, we selected candidate genes by PSI-BLAST search using Pvg3 as a query. Two homologous proteins were selected (CNAG_01050 and CNAG_01385). CNAG_01385 is Ggt1––an α-mannoside β-(1 → 6)-galactosyltransferase involved in GIPC biosynthesis in *C. neoformans*. Then, a BLASTp search was performed using CNAG_01050 as a query to select candidate genes. CNAG_06918 was selected in addition to CNAG_01385. Therefore, we named CNAG_01050 and CNAG_06918 as Ggt2 and Ggt3, respectively, the two α-galactoside β-(1 → 3)-galactosyltransferase candidates. Ggt2 and Ggt3 are members of the GT31 family. Ggt2 has an amino acid sequence homology of 23% (in the 434–645 amino acid region of Ggt1) and 33% (in the 122–320 amino acid region of Ggt3) with Ggt1 and Ggt3, respectively (Figure 1). Ggt1 does not have a transmembrane domain, but analysis using DeepLoc 2.0 (Thumuluri et al., 2022) predicted a transmembrane region at 338–360 aa. Ggt2 and Ggt3 were predicted to be Golgi-localized type II membrane proteins with one transmembrane region at N-terminal 35–57 and 21–43 aa, respectively, according to DeepLoc 2.0 (Figure 1).

### 3.2 ^1^H-NMR analysis of GXMGal from *GGT* mutants

**Figure 2:**
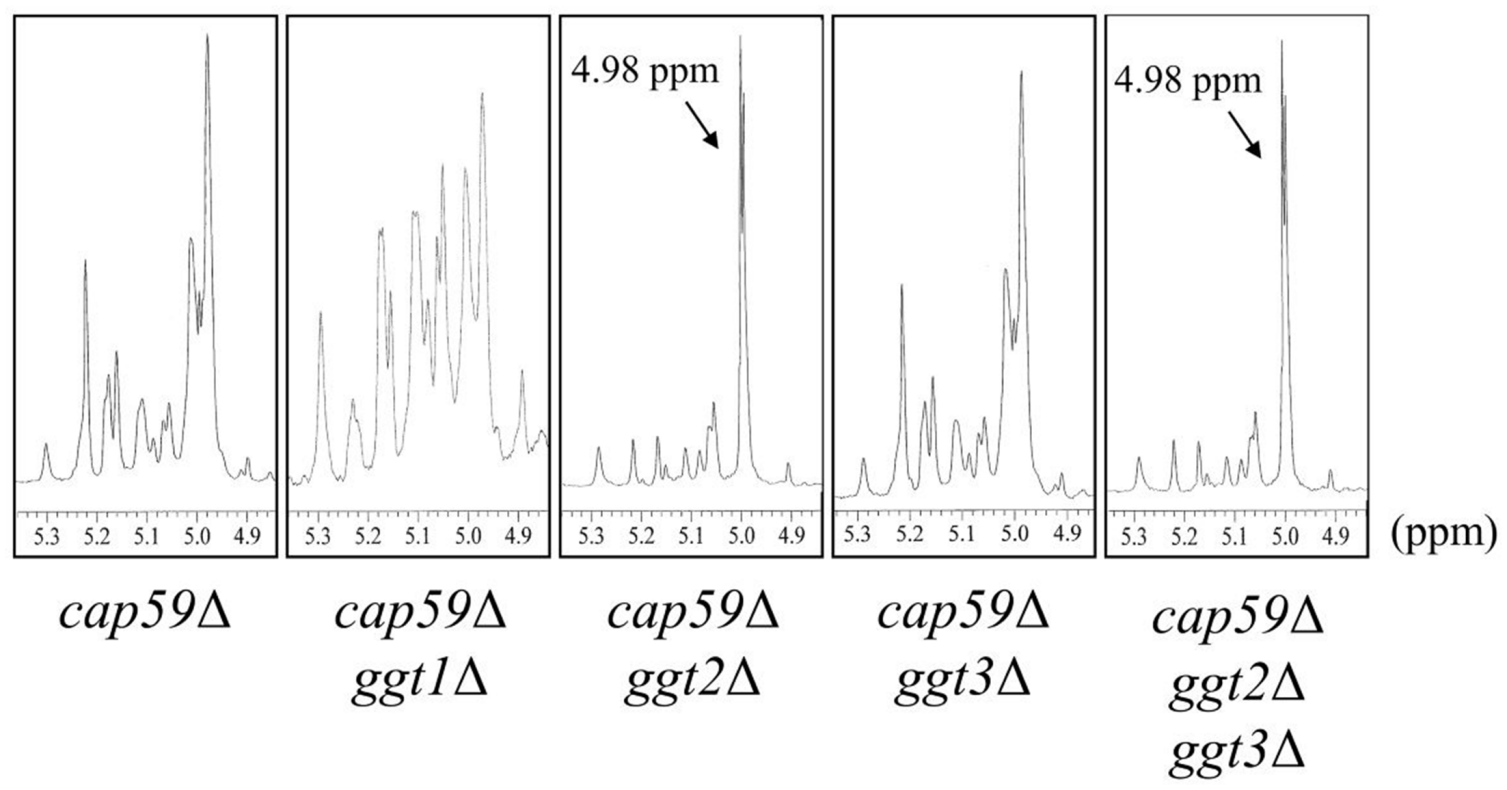
^1^H-NMR analysis of GXMGal from *cap59*Δ, *cap59*Δ *ggt1*Δ, *cap59*Δ *ggt2*Δ, *cap59*Δ *ggt3*Δ, and *cap59*Δ *ggt2*Δ *ggt3*Δ strains. ^1^H-NMR signals at 5.22, 5.16, and 4.99 ppm in *cap59*Δ, *cap59*Δ *ggt1*Δ, and *cap59*Δ *ggt3*Δ are derived from H-1 at position C-1 of the underlined Man residues in -(1 → 3)-Manα- and terminal Manα and the -(1 → 3)- Manα-(2 ← 1)- side chain of GXMGal. ^1^H-NMR signals at 4.98 ppm in *cap59*Δ *ggt2*Δ and *cap59*Δ *ggt2*Δ *ggt3*Δ are derived from H-1 at position C-1 of the underlined Gal residues in -(1 → 6)-Galα-. The proton chemical shifts were referenced relative to internal acetone at δ 2.225 ppm.

To investigate the effect of loss of presumptive GT31 family glycosyltransferases on GXMGal biosynthesis, we constructed *GGT1*, *GGT2*, and *GGT3* triple disruptants and *GGT2* and *GGT3* double disruptants using *cap59*Δ as parental strain. All strains were cultured in 10% Sabouraud medium for 5 days, and GXMGal was purified from the culture supernatant. Purified GXMGal was analyzed by 1H-NMR (Figure 2). Chemical shifts at ~5.22, 5.16, and 4.99 ppm in *cap59*Δ, *cap59*Δ*ggt1*Δ, and *cap59*Δ*ggt3*Δ strains indicated -(1 → 3)-Manα-, terminal Manα, and -(1 → 3)-Manα-(2 ← 1)-galactomannan side chain of GXMGal, respectively (Klutts and Doering, 2008). In *cap59*Δ*ggt2*Δ and *cap59*Δ*ggt2*Δ*ggt3*Δ, chemical shifts appeared at 4.98 ppm, indicating the presence of the -(1 → 6)-Galα- side chain. These results indicate that loss of *GGT2* results in a reduction or loss of the galactomannan side chain of GXMGal.

### 3.3 Phenotypic analysis of *GGT* mutants

**Figure 3:**
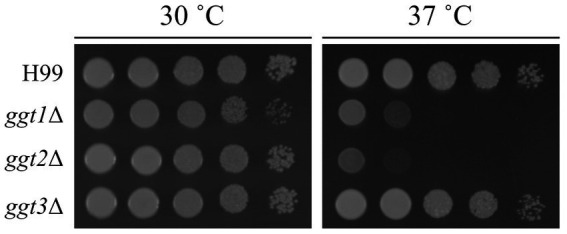
Phenotype of *ggt1*, *ggt2*, and *ggt3* single disruptants. Colony morphology of H99, *ggt1*Δ, *ggt2*Δ, and *ggt3*Δ cultured on YPD agar at 30°C and 37°C for 3 days, respectively. The agar medium was inoculated with 10-fold serial dilutions of cells adjusted to 10^6^ cells.

To clarify the physiological role of *GGT* in *C. neoformans* cells, *ggt1*, *ggt2*, and *ggt3* single-disruptant strains were constructed using H99 as the parental strain. As *uge1*Δ and *ugt1*Δ cannot supply UDP-Gal to the Golgi, they are deficient in GXMGal and GIPC biosynthesis and exhibit a Ts phenotype at 37°C (Moyrand et al., 2008; Li et al., 2017). *ggt1*Δ lacks GIPC and exhibits a Ts phenotype at 37°C. Therefore, we observed growth of *GGT* disruptants at 37°C (Figure 3). At 30°C, ggt1Δ showed slightly delayed growth compared with the wild-type; moreover, ggt2Δ and ggt3Δ showed similar growth compared with the H99 strain. By contrast, at 37°C, *ggt1*Δ and *ggt2*Δ showed dramatically delayed growth, indicating a Ts phenotype. The growth of *ggt3*Δ and H99 was similar. These results indicate that the glycan structure synthesized by Ggt2 is important for high-temperature stress tolerance in *C. neoformans*.

### 3.4 Drug resistance of *ggt2* disruptant strain

**Figure 4:**
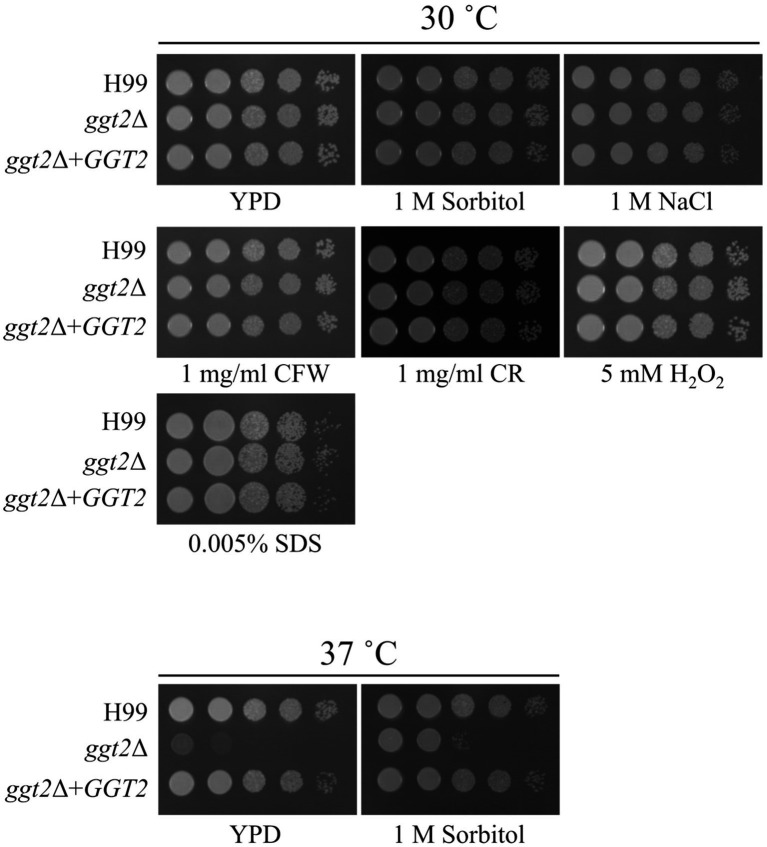
Drug sensitivity of the *ggt2* disruptant. Colony morphology of H99, *ggt2*Δ, and *ggt2*Δ + *GGT2* on YPD agar supplemented with or without 1 M sorbitol, 1 M NaCl, 1 mg/mL calcofluor white (CFW), 1 mg/mL Congo red (CR), 5 mM H_2_O_2_, and 0.005% sodium dodecyl sulfate (SDS) at 30°C and 37°C for 3 days. The agar medium was inoculated with 10-fold serial dilutions of cells adjusted to 10^6^ cells.

We examined the growth of *ggt2*Δ on media containing various drugs. *ugt1* disruptant strains show sensitivity to NaCl, Congo red, H_2_O_2_, and sodium dodecyl sulfate (SDS) (Li et al., 2017). Therefore, we tested the sensitivity of *ggt2*Δ to these drugs (Figure 4). *ggt2*Δ was not significantly sensitive to any of the drugs. Conversely, the Ts phenotype of *ggt2*Δ was completely rescued by complementation with wild-type *GGT2* and slightly rescued by 1 M sorbitol, which exerts high osmotic pressure. These results indicate that the galactomannan side chain of GXMGal biosynthesized by Ggt2 is important for high-temperature stress tolerance in *C. neoformans*.

### 3.5 Capsule productivity of *ggt2* disruptant strain

To further analyze the *ggt2*Δ phenotype, the capsule structure was stained with India ink for microscopic observation (Supplementary Figure S3). Quantification of cell and capsule sizes revealed no significant differences between *ggt2*Δ and the wild-type strain. These findings indicated that Ggt2 absence did not affect GXM production.

### 3.6 Role of Ggt2 in GXMGal biosynthesis

**Figure 5:**
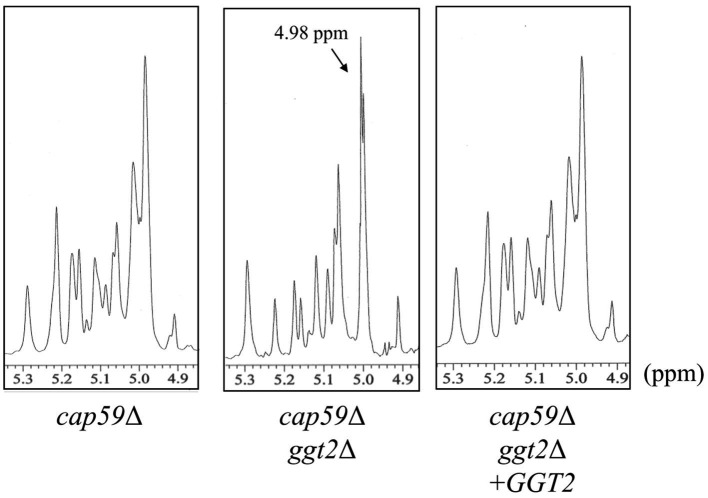
^1^H-NMR analysis of GXMGal from *cap59*Δ, *cap59*Δ *ggt2*Δ, and *cap59*Δ *ggt2*Δ + *GGT2* strains. ^1^H-NMR signals at 5.22, 5.16, and 4.99 ppm in *cap59*Δ and *cap59*Δ *ggt2*Δ + *GGT2* are derived from H-1 at position C-1 of the underlined Man residues in -(1 → 3)-Manα- and terminal Manα and the -(1 → 3)- Manα-(2 ← 1)- side chain of GXMGal. ^1^H-NMR signal at 4.98 ppm in *cap59*Δ*ggt2*Δ is derived from H-1 at position C-1 of the underlined Gal residues in -(1 → 6)-Galα-. The proton chemical shifts were referenced relative to internal acetone at δ 2.225 ppm.

To examine the role of Ggt2 in GXMGal biosynthesis, we complemented wild-type *GGT2* in *cap59*Δ*ggt2*Δ and analyzed the structure of GXMGal. The structure of GXMGal produced by each strain was analyzed by ^1^H-NMR (Figure 5). Sharp doublet peaks at 4.98 ppm of the α-galactan backbone seen in *cap59*Δ*ggt2*Δ disappeared due to *GGT2* complementation, and the Man-derived chemical shift seen in wild-type GXMGal reappeared.

Methylation GC–MS analysis was used to investigate constituent sugars and the linkage mode of GXMGal produced by each strain (Table 1). In GXMGal produced by *cap59*Δ*ggt2*Δ, terminal Xyl (tXyl_1_) residues were reduced to below the detection limit. Additionally, 4-substituted Gal (_4_Gal_1_) and 3,4-substituted Gal (_3,4_Gal_1_) (indicating β-Gal residues in the galactomannan side chain) and 3,6-substituted Gal (_3,6_Gal_1_) (indicating α-Gal residues attached to galactomannan side chains) were reduced to below detection limit. The ratio of 2,6-substituted Gal (indicating α-Gal with Gal*f* side chain) and terminal Gal (tGal_1_) and Gal*f* (tGal*f*_1_) increased. By contrast, the ratio of 3-substituted Gal (_3_Gal_1_) and 6-substituted Man (_6_Gal_1_), which are not present in GXMGal, increased drastically. Terminal Man (tMan_1_), 3-substituted Man (_3_Man_1_), and 2,3-substituted Man (_2,3_Man_1_) decreased but did not disappear.

**Table 1:** Methylation analysis of GXMGal from the *ggt2* disruptant.

	Mol %		
Residues*^a^*	*cap59*Δ	*cap59*Δ*ggt2*Δ*^b^*	*cap59*Δ*ggt2*Δ + *GGT2*
tXyl_1_	11.9	n.d.	12.67
tMan_1_	16.3	11.79	17.96
tGalp_1_	3.02	7.11	2.87
tGal*f*_1_	1.55	5.8	2.25
_2_Man_1_	7.61	7.62	6.92
_4_Gal_1_	7.8	n.d.	6.26
_3_Man_1_	8.83	3.09	10.09
_6_Man_1_	2.35	3.84	4.47
_3_Gal_1_	13.03	47	12.49
_3,4_Gal_1_	12.08	n.d.	9.75
_2,3_Man_1_	2.5	4.94	2.66
_6_Gal_1_	9.73	3.77	6.55
_2,6_Gal_1_	1.88	5.03	3.97
_3,6_Gal_1_	1.41	n.d.	1.92

^a^t means non-reducing terminal.

^b^n.d. means none detected.

**Figure 6:**
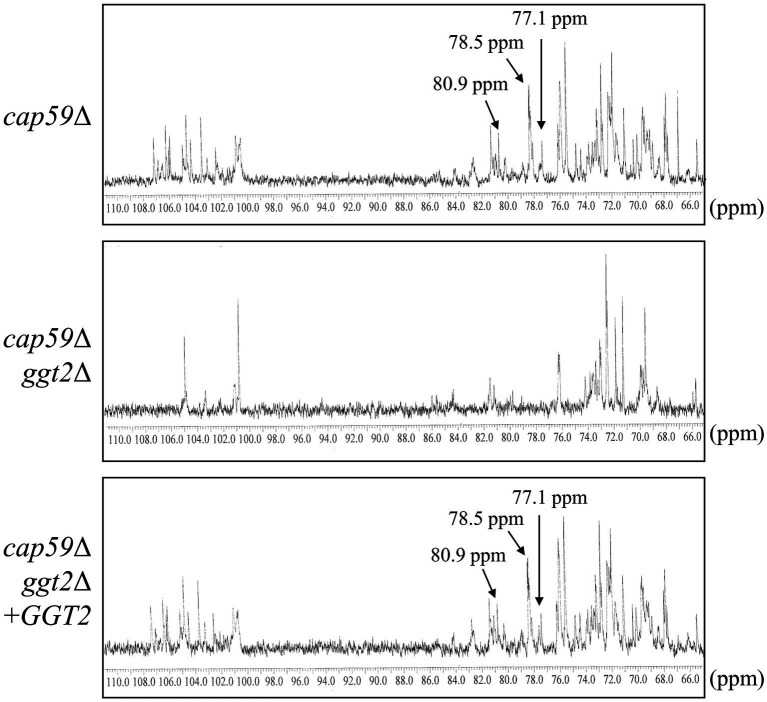
^13^C-NMR analysis of GXMGal from *cap59*Δ, *cap59*Δ *ggt2*Δ, and *cap59*Δ *ggt2*Δ + *GGT2* strains. Broad chemical signals at 100.6–105.1 ppm in *cap59*Δ and *cap59*Δ *ggt2*Δ + *GGT2* are derived from position C-1 of Manα-(1 → 3)-, 2,3-*O*-substituted-Manα, Xylβ-(1 → 2)-, and Xylβ-(1 → 3)- in the galactomannan side chain. ^13^C-NMR signals at 78.5 and 77.1 ppm in *cap59*Δ and *cap59*Δ *ggt2*Δ + *GGT2* are derived from position C-4 of Gal residues in 4-substituted Galβ and the 3,4-substituted Galβ side chain of GXMGal. The ^13^C-NMR signal at 80.9 ppm in *cap59*Δ and *cap59*Δ *ggt2*Δ + *GGT2* is derived from position C-3 of Gal residues in 3,6-substituted Galα. The carbon chemical shifts were referenced relative to internal acetone at δ 31.07 ppm.

We analyzed the structure of GXMGal in detail by ^13^C-NMR (Figure 6). In GXMGal from *cap59*Δ, we detected a broad chemical shift at 100.6–105.1 ppm, possibly derived from position C-1 of Manα-(1 → 3)-, 2,3-*O*-substituted-Manα, Xylβ-(1 → 2)-, and Xylβ-(1 → 3)- in the galactomannan side chain (Klutts and Doering, 2008). These structures were suggested to be lost with the loss of *GGT2*. The chemical shifts at 78.5 and 77.1 ppm, which originate from position C-4 of 4-substituted Galβ and 3,4-substituted Galβ in wild-type GXMGal, disappeared in *ggt2Δ*. The chemical shift at 80.9 ppm originating from position C-3 of 3,6-substituted Galα disappeared in *ggt2Δ*. These results strongly support our hypothesis that Ggt2 catalyzes β-(1 → 3)-Gal transfer to α-galactoside––the initial reaction in the biosynthesis of the galactomannan side chain of GXMGal.

### 3.7 Phylogenetic analysis of Pvg3, Ggt1, Ggt2, and Ggt3 family proteins belonging to the GT31 family

**Figure 7:**
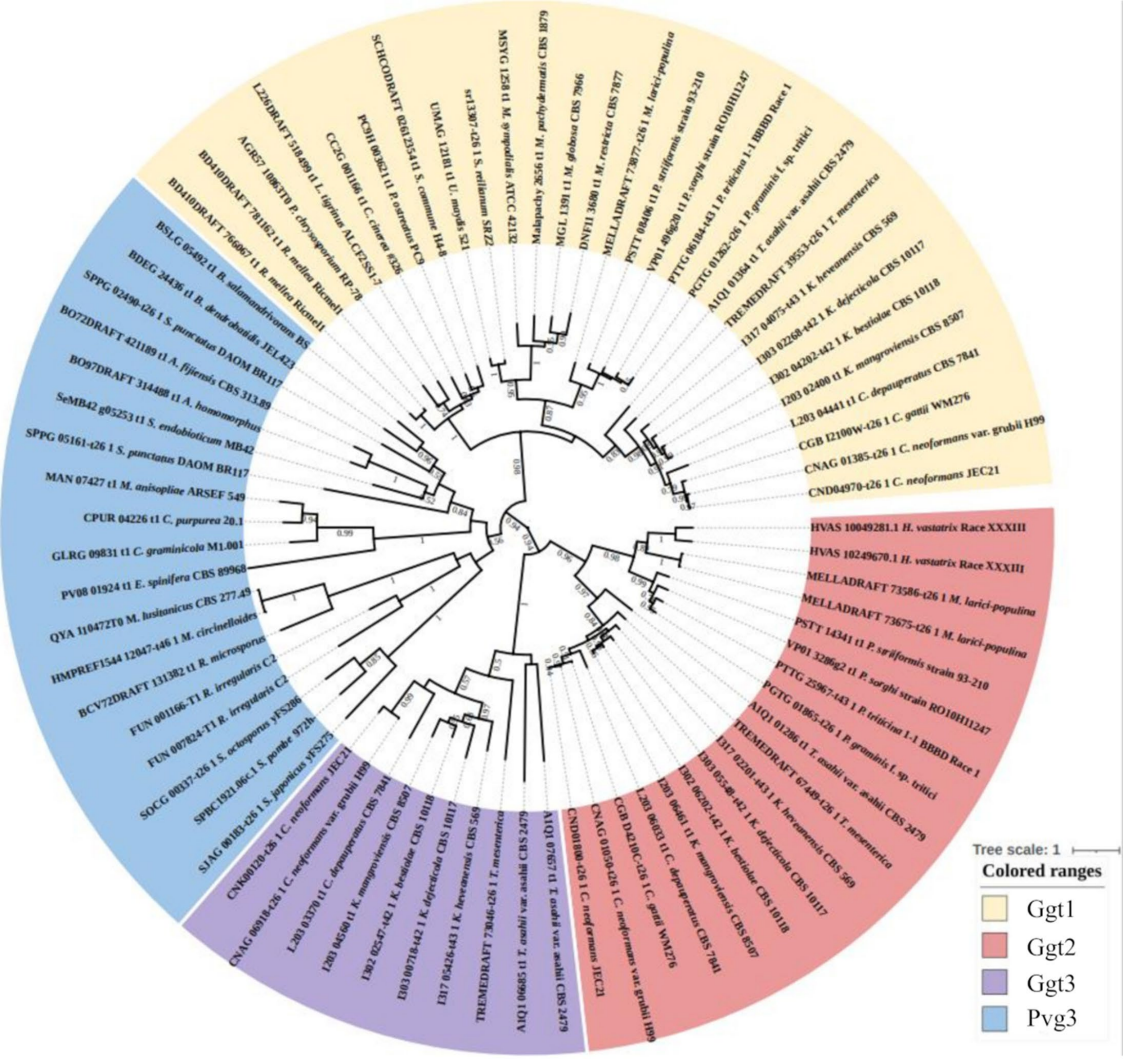
Phylogenetic analysis of Ggt1, Ggt2, and Ggt3 homologs in basidiomycetes and Pvg3 homologs in basidiomycetes and fission yeasts. Protein sequences were downloaded from FungiDB. The phylogenetic tree was drawn using iTOL. Alignment and phylogenetic tree inference were performed using MAFFT and RAxML, respectively, included in ETE v3.

Sequences of Pvg3, Ggt1, Ggt2, and Ggt3 family proteins were used to construct an evolutionary phylogenetic tree (Figure 7). The data set for analysis was obtained from FungiDB using the amino acid sequences of *S. pombe* Pvg3 and *C. neoformans* Ggt1, Ggt2, and Ggt3 as search queries.^1^ The protein sequences were clearly divided into the Pvg3, Ggt1, Ggt2, and Ggt3 clades (Figure 7). Ggt1 was widely distributed in Basidiomycota, including *Ustilago*, *Coprinopsis*, *Schizophyllum*, and *Cryptococcus*. Ggt2 was distributed in Pucciniomycetes and Tremellomycetes in Basidiomycota. Furthermore, Ggt3 was distributed only in Tremellomycetes in Basidiomycota.

## 4 Discussion

**Figure 8:**
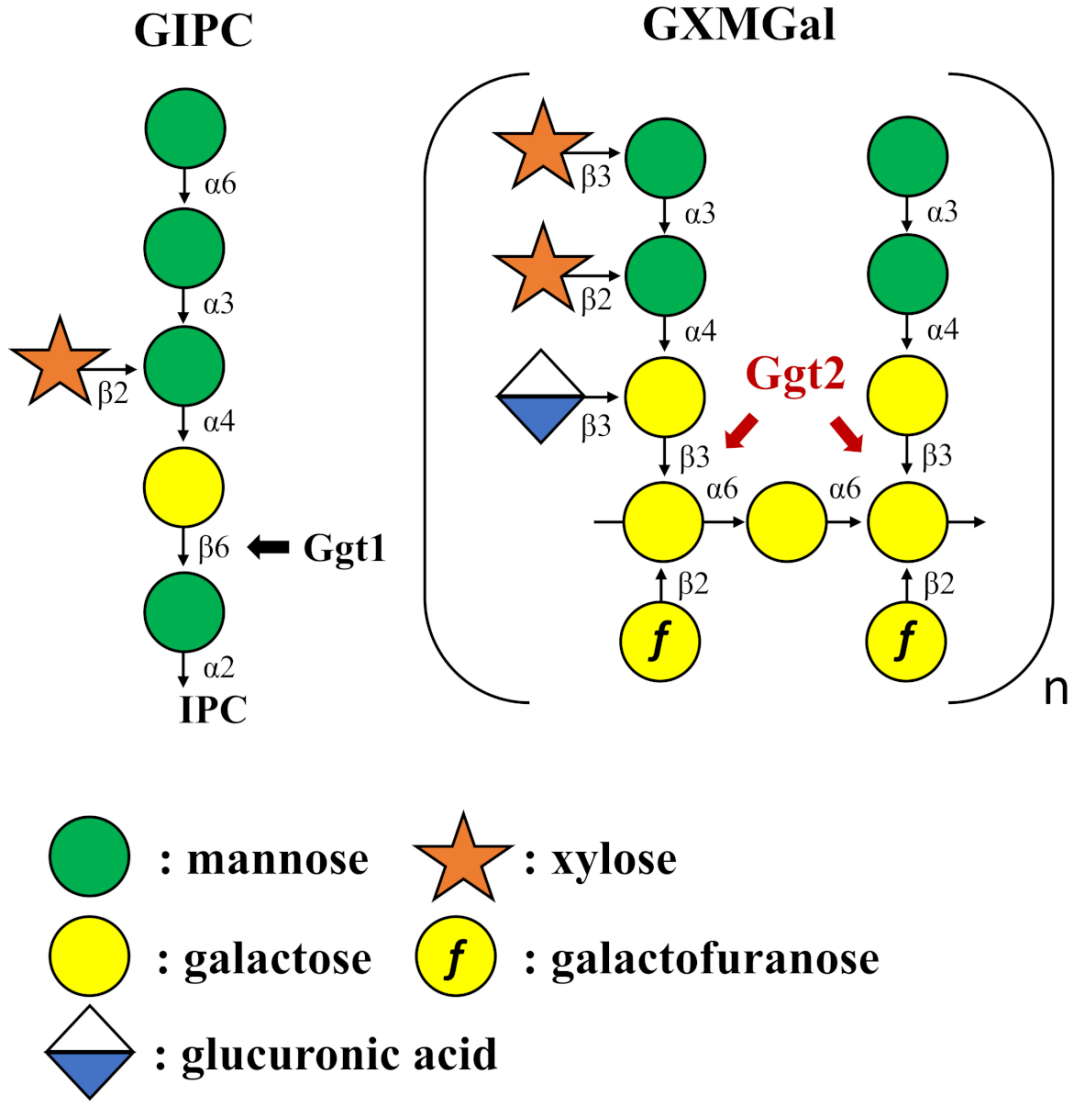
Summary model of GIPC and GXMGal biosynthesis in *Cryptococcus neoformans*. Ggt1 represents GIPC α-mannoside β-(1 → 6)-galactosyltransferase. Ggt2 represent α-galactoside β-(1 → 3)-galactosyltransferase.

This study aimed to identify glycosyltransferases involved in the biosynthesis of *C. neoformans* capsules. We identified a putative β-(1 → 3)-Gal transferase belonging to the GT31 family that plays a role in GXMGal biosynthesis (Figure 8). Ggt1 is conserved in a wide range of species in the phylum Basidiomycota, whereas Ggt2 is only conserved in certain basidiomycete yeasts, such as Pucciniomycetes and Tremellomycetes (Figure 7). Multiple alignments of Ggt2 homologs revealed that amino acids in the GT-A domain are highly conserved (Supplementary Figure S4). Therefore, Ggt2 homologs may be responsible for synthesizing important glycan structures, including GXMGal. The enzyme involved in capsule biosynthesis has attracted attention as a novel antifungal drug target due to its contribution to virulence (Almeida et al., 2015). Many putative glycosyltransferases, involved in GXM biosynthesis, have been identified as *CAP* genes, because they can be easily screened based on phenotypes, such as India ink-negative staining (Fromtling et al., 1982). However, identification of glycosyltransferases involved in capsule biosynthesis is challenging, and only a few glycosyltransferases have been identified: α-(1 → 3)-Man transferase Cmt1 (Doering, 1999; Sommer et al., 2003) is involved in GXM biosynthesis and β-(1 → 2)-Xyl transferases Cxt1 and Cxt2 are involved in GXMGal biosynthesis (Klutts et al., 2007; Klutts and Doering, 2008; Reilly et al., 2009; Wang et al., 2018). We believe that our discovery of Ggt2 will contribute to studies on capsule biosynthesis in *C. neoformans.*

Methylation GC–MS and NMR analyses revealed the detailed role of Ggt2 in GXMGal biosynthesis (Table 1; Figure 6). The loss or severe reduction of the galactomannan side chain of GXMGal in the *ggt2* disruptant strain indicates that Ggt2 is the only α-Gal β-(1 → 3)-Gal transferase involved in GXMGal biosynthesis (Figure 2) because Ggt1 and Ggt2 accept α-mannoside and α-galactoside as receptor substrates, respectively. This is a logical result because the structure of the sugar chains involved in their biosynthesis suggests that Ggt1 and Ggt2 are likely to use α-mannoside and α-galactoside as their acceptor substrates, respectively. The GXMGal structure between the *ggt3*Δ and wild-type strains was not notably different and did not exhibit a Ts phenotype, suggesting that Ggt3 is not involved in GXMGal or GIPC biosynthesis (Figures 3, 4). The function of Ggt3 must be analyzed in detail. Interestingly, methylation GC–MS analysis detected methyl-esterified sugars that may have originated from glycans other than GXM and GXMGal. These indicate the presence of unknown glycan structures, such as *N*- or *O*-glycans or glycolipids. Considering how GXMGal was first discovered in the culture supernatant of a mutant strain lacking GXM (Cherniak et al., 1982), *C. neoformans may* possess unknown glycan structures. Thus, the detailed structures of these glycans should be clarified in the future.

Phenotypic analysis of gene disruptant strains revealed the physiological functions of Ggt2. *ggt2*Δ, like *ggt1*Δ, exhibited a Ts phenotype at 37°C, indicating that the galactomannan side chain of GXMGal, like GIPC, is important for high-temperature stress tolerance in *C. neoformans*. Notably, GXMGal is important for high-temperature stress tolerance in *C. neoformans*, although it is less abundant than GXM. In *C. neoformans*, *Saccharomyces cerevisiae*, and *Aspergillus fumigatus*, *O*-glycan deficiency leads to reduced high-temperature stress tolerance and disruption of cell wall integrity (Wagener et al., 2008; Kadooka et al., 2022; Thak et al., 2022). However, *ggt2*Δ was insensitive to Congo red or calcofluor white, which are inhibitors of cell wall synthesis, suggesting that loss of galactomannan side chains in GXMGal does not affect cell wall integrity in *C. neoformans*. Additionally, *ugt1*Δ is sensitive to Congo red, NaCl, and SDS (Li et al., 2017), but *ggt*2Δ is not, suggesting that the phenotype of *ugt1*Δ is not due to the loss of galactomannan side chains of GXMGal but rather due to a loss of GIPC. Consistently, GXM-deficient mutants were sensitive to NaCl and SDS (Li et al., 2018a,b). The phenotypic differences in polysaccharide-deficient mutants are interesting and should be analyzed further.

We used a bacterial heterologous expression system to generate recombinant Ggt2 and measured its β-Gal transfer activity to 4-methylumbelliferylated α-Gal. However, we could not detect glycosyltransfer activity (data not shown). This may be characteristic of the substrate specificity of Ggt2. The galactomannan side chain of GXMGal is added in succession, probably because Ggt2 recognizes di- or trisaccharide α-galacto-oligosaccharides as substrates and can only transfer β-Gal to a certain location. Another hypothesis is that Ggt2 does not exhibit glycosyltransferase activity by itself. For example, *S. pombe* Pvg3, which belongs to the GT31 family, is not active alone but exhibits glycosyltransferase activity by forming a complex with several proteins (Fukunaga et al., 2023). The enzymatic features of Ggt2 may be essential for the formation of the unique GXMGal structure and should be studied in more detail.

In conclusion, we have partially identified the mechanism of GXMGal biosynthesis in *C. neoformans*. Our findings will contribute substantially to understanding the structure and biosynthesis of the fungal cell wall and developing anticryptococcal agents.

## Data availability statement

The datasets presented in this study can be found in online repositories. The names of the repository/repositories and accession number(s) can be found in the article/Supplementary material.

## Author contributions

CK: Writing – original draft, Writing – review & editing, Conceptualization, Data curation, Formal analysis, Funding acquisition, Investigation, Project administration. YT: Writing – review & editing, Data curation, Methodology, Validation. DH: Writing – review & editing, Data curation, Methodology, Validation. TO: Writing – original draft, Writing – review & editing, Conceptualization, Data curation, Formal analysis, Funding acquisition, Investigation, Project administration, Supervision.

## Footnotes

^1^ https://fungidb.org

## Supplementary material

### SUPPLEMENTARY FIGURE S1

Construction of *Cryptococcus neoformans* gene disruption strains. **(A)** Chromosomal maps of *cap59, ggt1, ggt2,* and *ggt3* disruptant strains. The positions of primers are indicated by arrows. **(B)** Confirmation of correct recombination of each strain using PCR analysis. Electrophoretic analysis of products amplified by PCR using the xxxX-comf-F–xxxX-comf-R primer pair. M, DNA size markers (Gene Ladder Wide 2; Nippon Gene, Tokyo, Japan).

### SUPPLEMENTARY FIGURE S2

Construction of *GGT2* complementation strains. **(A)** Chromosomal map of ggt2 disruptant strain. The positions of primers are indicated by arrows. **(B)** Confirmation of correct recombination of each strain using PCR analysis. Electrophoretic analysis of products amplified by PCR using the ggt2-comp-comf-F–ggt2-comp-comf-R primer pair. M, DNA size markers (Gene 493 Ladder Wide 2; Nippon Gene, Tokyo, Japan).

### SUPPLEMENTARY FIGURE S3

Visualization of capsular structure. **(A)** Capsule imaging of H99, *ggt2Δ, ggt2Δ+GGT2*, and *cap59Δ*. *C. neoformans* strains were pre-cultured in YPD liquid medium at 30°C for 24 h, transferred to 10% Sabouraud liquid medium and further cultured at 30°C for 24 h. Finally, the cellswere stained with India ink and observed microscopically. Scale bars: 10 μm. **(B)** Capsule size quantification. The average diameter was calculated for the cells and capsules of H99, *ggt2Δ, ggt2Δ+GGT2*, and *cap59Δ*.

### SUPPLEMENTARY FIGURE S4

Multiple sequence alignment of Ggt2 homologs. Multiple sequence alignment was performed using MAFFT v6.864 in GenomeNet Bioinformatics Tools and displayed in ESPript 3.0
