# Supplementary material for: Identification of a putative α-galactoside β-(1 → 3)-galactosyltransferase involved in the biosynthesis of galactomannan side chain of glucuronoxylomannogalactan in Cryptococcus neoformans
Source: Front Microbiol. 2024 May 22;15:1390371. doi: 10.3389/fmicb.2024.1390371 (PMC11150766; doi:10.3389/fmicb.2024.1390371)
Supplement: Supplementary file 2 [file Presentation_1.PPTX]

## Slide 1
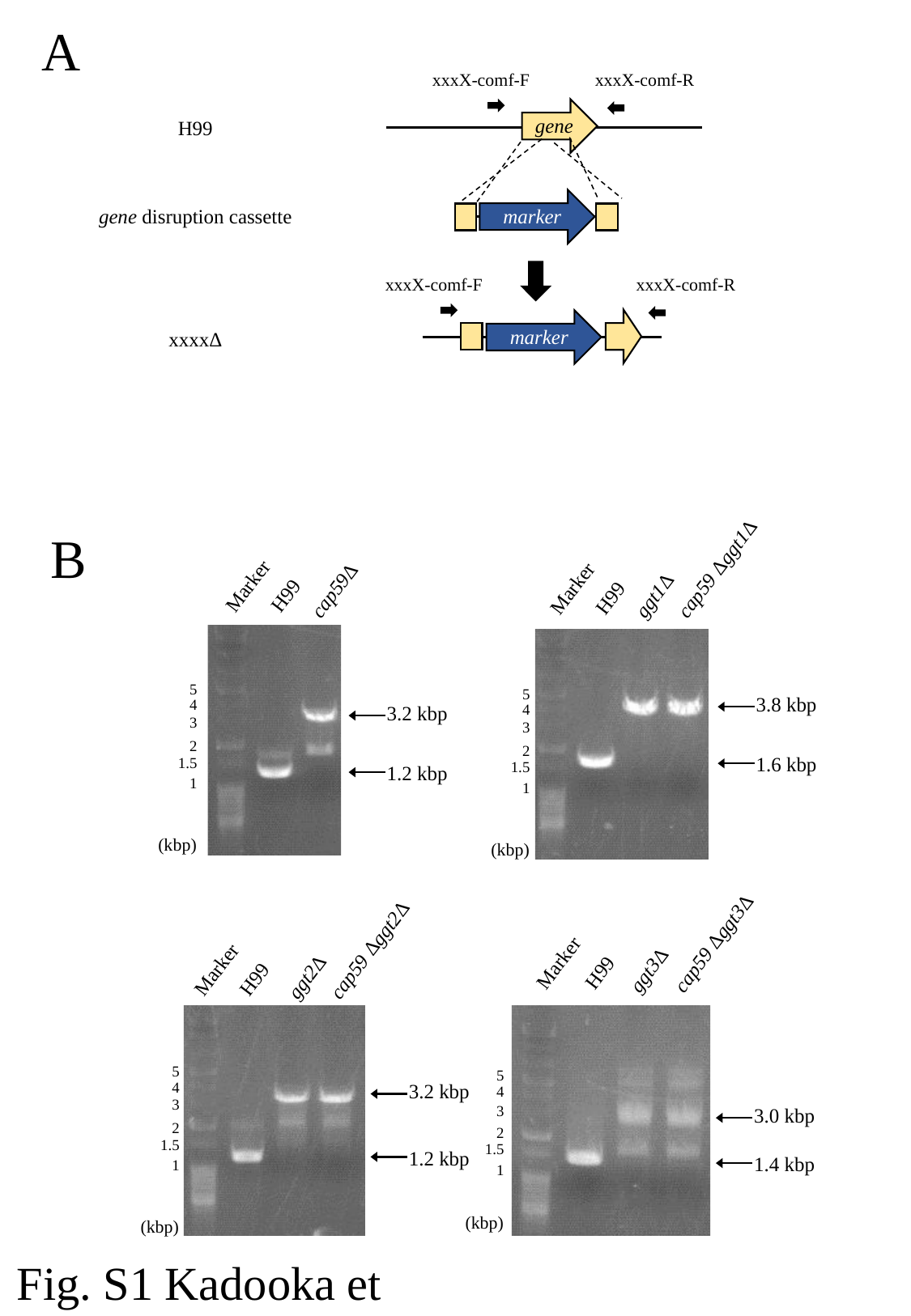

A
xxxX-comf-F
xxxX-comf-R
gene
H99
marker
gene disruption cassette
xxxX-comf-F
xxxX-comf-R
marker
xxxxΔ
B
cap59 Δggt1Δ
Marker
H99
Marker
H99
ggt1Δ
cap59Δ
5
5
3.8 kbp
4
4
3.2 kbp
3
3
2
2
1.6 kbp
1.5
1.5
1.2 kbp
1
1
(kbp)
(kbp)
cap59 Δggt3Δ
Marker
H99
cap59 Δggt2Δ
ggt3Δ
Marker
H99
ggt2Δ
5
5
4
3.2 kbp
4
3
3
3.0 kbp
2
2
1.5
1.5
1.2 kbp
1.4 kbp
1
1
(kbp)
(kbp)
Fig. S1 Kadooka et al.

## Slide 2
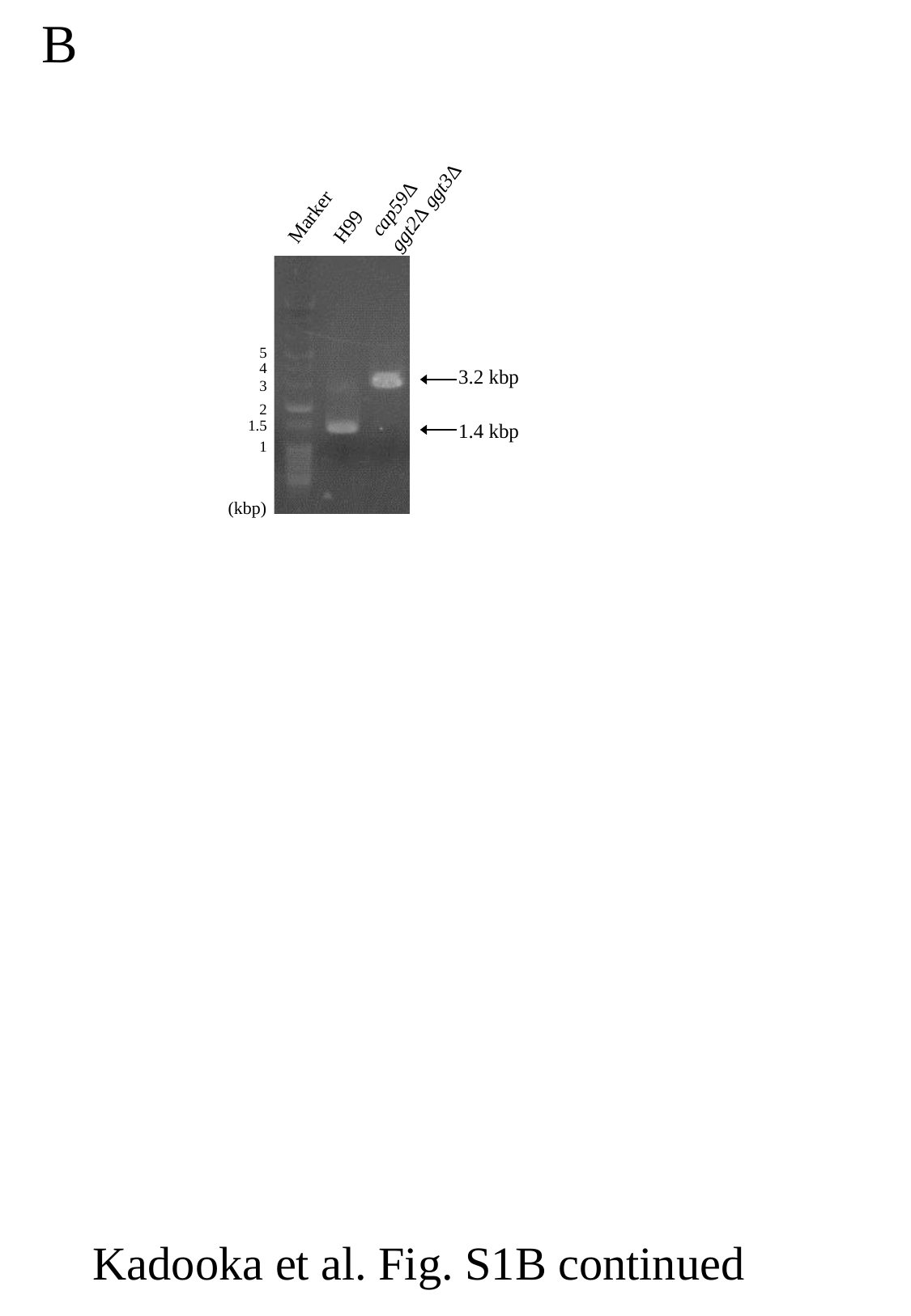

B
cap59Δ ggt2Δ ggt3Δ
Marker
H99
5
4
3.2 kbp
3
2
1.5
1.4 kbp
1
(kbp)
Kadooka et al. Fig. S1B continued

## Slide 3
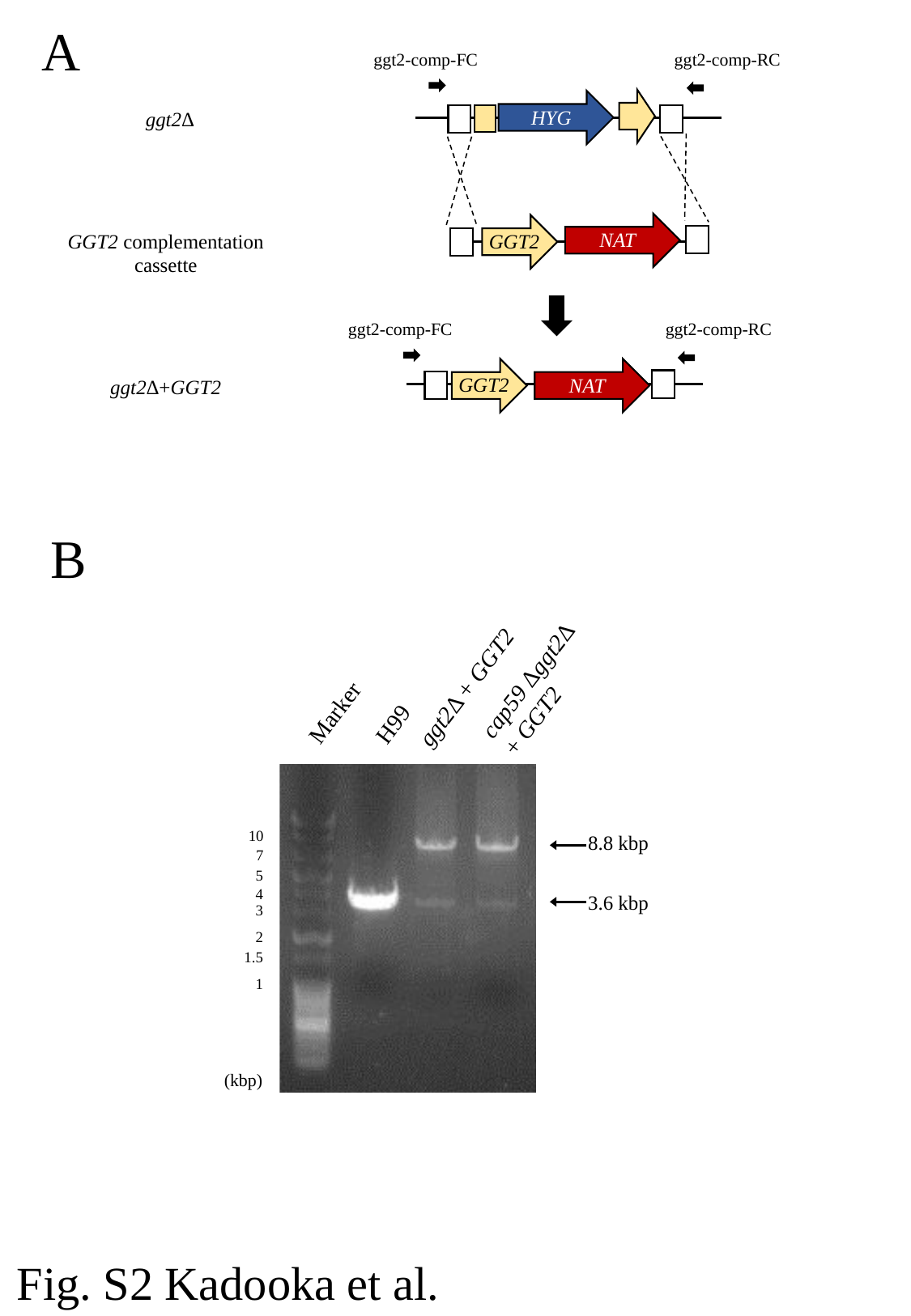

A
ggt2-comp-FC
ggt2-comp-RC
HYG
ggt2Δ
NAT
GGT2 complementation cassette
GGT2
ggt2-comp-FC
ggt2-comp-RC
GGT2
NAT
ggt2Δ+GGT2
B
cap59 Δggt2Δ
+ GGT2
ggt2Δ + GGT2
Marker
H99
10
8.8 kbp
7
5
4
3.6 kbp
3
2
1.5
1
(kbp)
Fig. S2 Kadooka et al.

## Slide 4
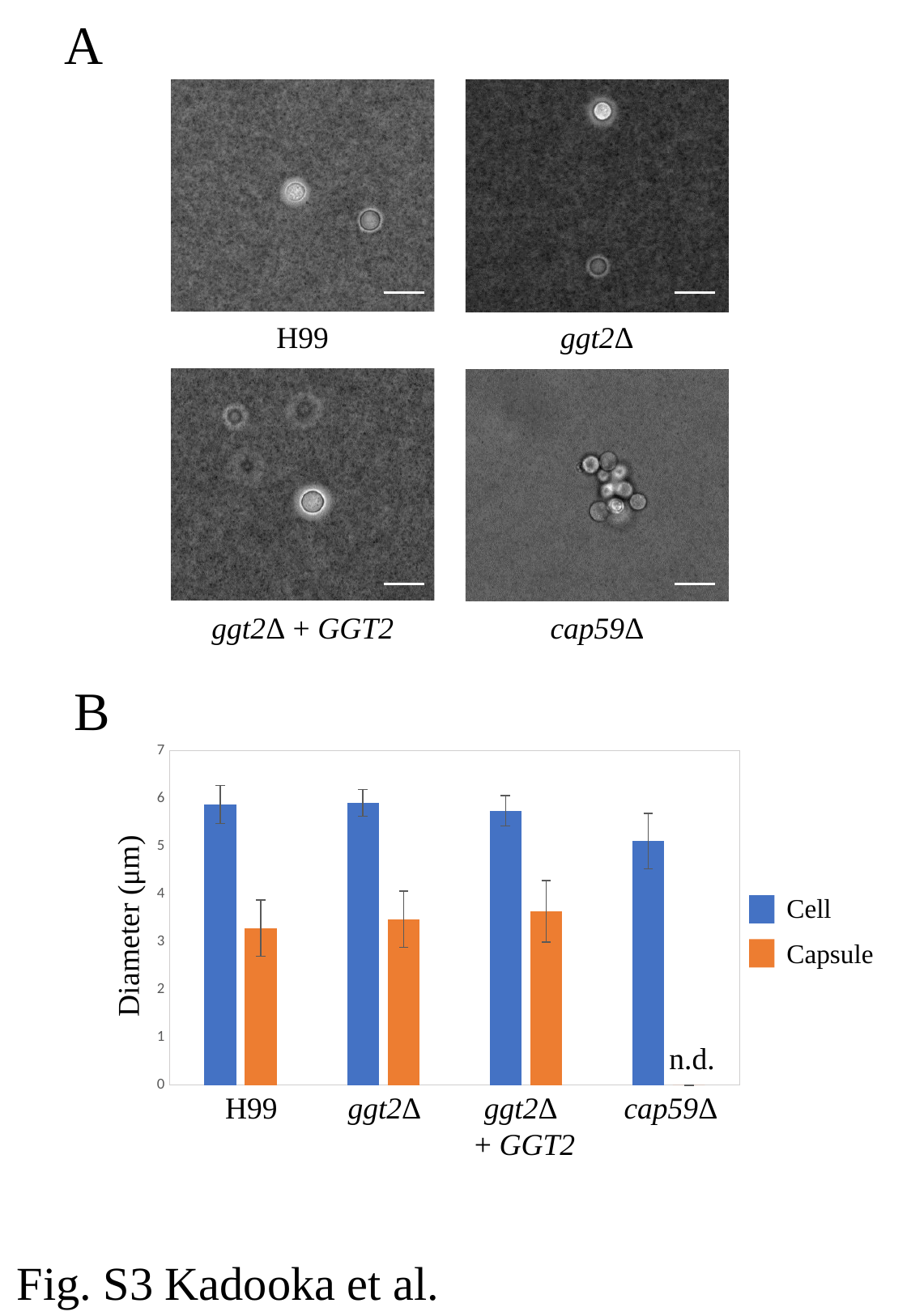

A
H99
ggt2Δ
ggt2Δ + GGT2
cap59Δ
B
### Chart
| Category | | |
|---|---|---|Cell
Diameter (μm)
Capsule
n.d.
H99
ggt2Δ
ggt2Δ
+ GGT2
cap59Δ
Fig. S3 Kadooka et al.

## Slide 5
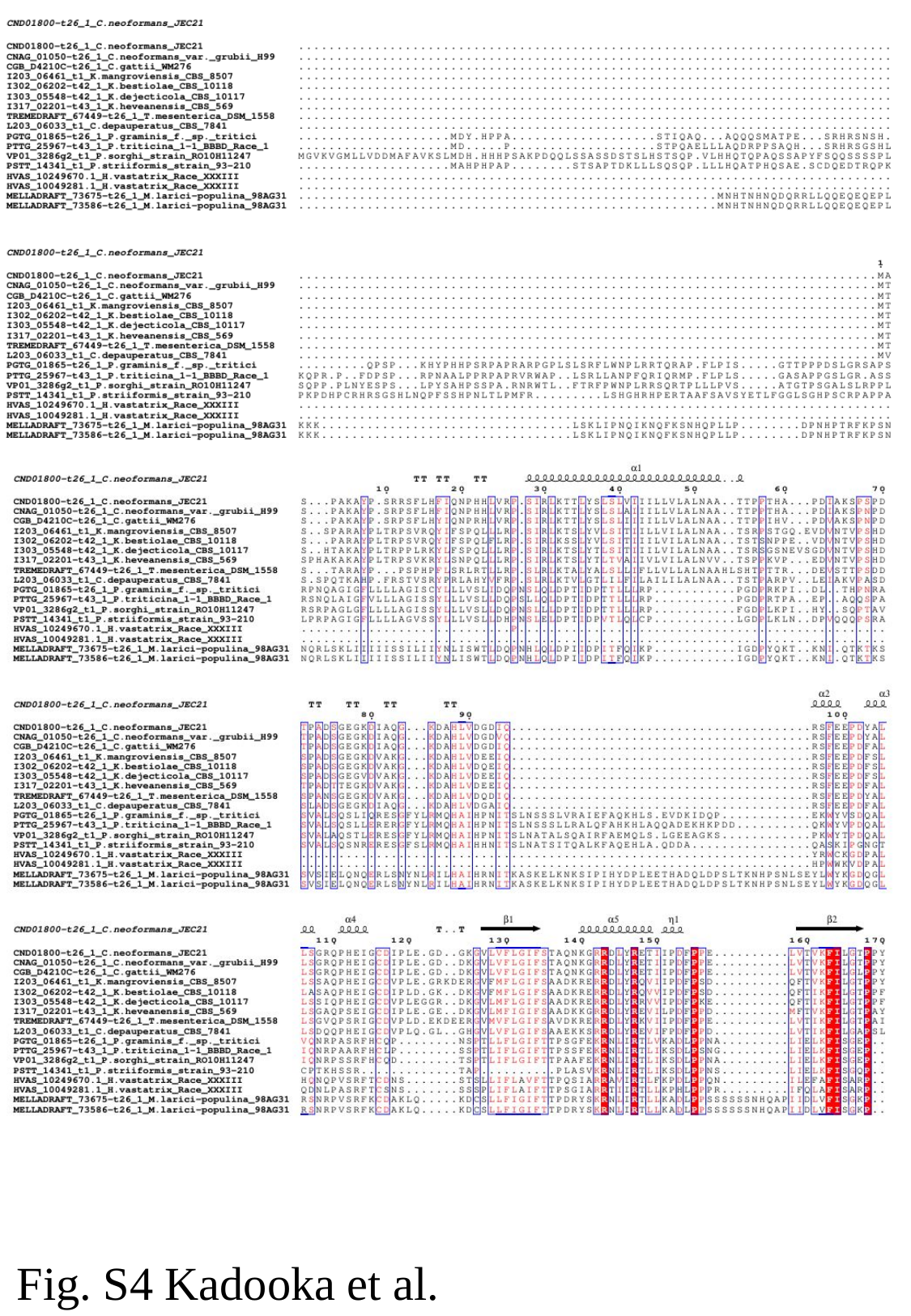

Fig. S4 Kadooka et al.

## Slide 6
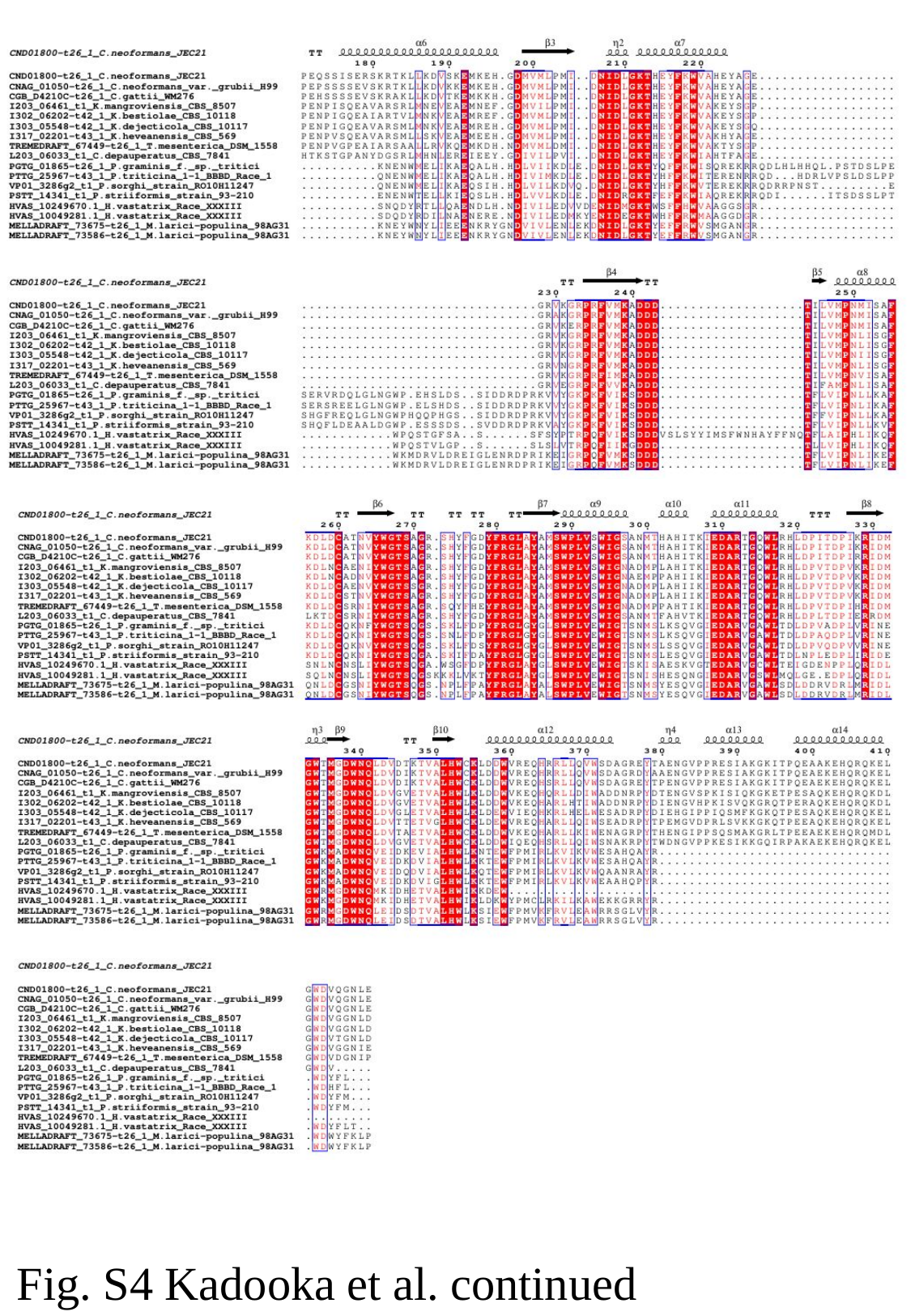

Fig. S4 Kadooka et al. continued
